# Preoperative oral antibiotics reduce infections after colorectal cancer surgery

**DOI:** 10.1007/s00423-016-1513-1

**Published:** 2016-09-20

**Authors:** Michal Mik, Maciej Berut, Radzislaw Trzcinski, Lukasz Dziki, Jaroslaw Buczynski, Adam Dziki

**Affiliations:** 1 Department of General and Colorectal Surgery, Medical University of Lodz, Plac Hallera 1, 90-647 Lodz, Poland; 2 Centre for Treatment of Bowel Diseases, Hospital in Brzeziny, Brzeziny, Poland; 3 Department of Nutrition, Medical University of Lodz, Lodz, Poland

**Keywords:** Colorectal cancer, Surgical site infection, Bowel preparation, Anastomotic leak, Oral antibiotic prophylaxis

## Abstract

**Aim:**

The objectives were to recognize the risk factors for surgical site infections (SSIs) after surgery due to colorectal cancer and to assess the impact of mechanical bowel preparation (MBP) and oral antibiotic prophylaxis (ABX) on SSIs.

**Methods:**

Records from two colorectal centers were used. Risk factors of SSIs were categorized into patient-, disease-, and treatment-dependent.

**Results:**

A group of 2240 patients was included. SSIs were noted in 364 patients (16.3 %). MBP+/ABX+ was connected with a lower incidence of anastomotic leak (AL) and organ-space SSIs: 2.4 vs. 6.3 %; *p* = 0.008 and 3.6 vs. 7.2 %; *p* = 0.017, respectively. Patient-dependent factors: obesity increased the risk of skin superficial SSIs, adjusted OR 1.53 (1.47–1.59 95 % confidence interval (95 % CI)), and deep incisional SSIs 1.42 (1.39–1.45 95 % CI). Disease-dependent factors: rectal cancer was associated with a higher risk of skin superficial and deep incisional SSIs, adjusted OR 1.28 (1.22–1.34 95 % CI) and 1.13 (1.09–1.15 95 % CI). Treatment-dependent factors: MBP+/ABX+ was associated with a lower risk of organ-space SSIs, adjusted OR 0.53 (0.44–0.59 95 % CI). Radiotherapy increased the risk of organ-space SSIs, adjusted OR 1.78 (1.75–1.80 95 % CI). The risk of organ-space SSIs was the highest after low anterior resection, adjusted OR 1.62 (1.60–1.64 95 % CI).

**Conclusions:**

If possible, MBP and ABX should always be administered to decrease the risk of AL and organ-space SSIs. Factors strictly related to the treatment mostly increased the risk of organ-space SSIs.

## Introduction

Surgical site infections (SSIs) are commonly diagnosed as postoperative complications related to all abdominal operations with an estimated rate of 26 %. In colorectal surgery, the rate ranges from 15 to 35 % [1, 2]. The occurrence of SSIs depends on many factors, such as the patient, the disease, the surgeon’s experience and surgical technique, mechanical bowel preparation, and antibiotic prophylaxis. Sometimes, it is difficult to anticipate which group of patients is at a higher risk of SSIs.

In the 1970s, the utilization of mechanical bowel preparation (MBP) and oral antibiotic prophylaxis (ABX) became a standard preoperative regimen [3]. Despite the accepted procedure, some practitioners question the use of the components of MBP since MBP does not protect from SSIs [4, 5] and some components of MBP may even be harmful [6]. During recent years, there has been intensified interest in the influence of MBP and ABX on the outcomes and the latest research has proven a renewed view [7, 8]. The data suggest that MBP+ along with ABX+ may reduce the incidence of SSIs compared with the strategy of MBP− and ABX− [9].

The Centers for Disease Control and Prevention (CDCP) distinguishes SSIs into three different types based on an anatomical level of infection: skin superficial, deep incisional, and organ-space [10]. These three particular types of SSIs may be related to different clinical risk factors, and the aggregation of these types seems to be improper and leads to negative implications and some bias for measurement of quality of care [11].

Skin superficial SSIs may not be serious and may be diagnosed mainly after discharge. Deep incisional and organ-space SSIs can be life threatening, and their treatment often involves certain costs. Many patients with deep and intraabdominal SSIs need readmission, urgent redo surgery, intravenous antibiotics, or percutaneous drainage [12]. Additionally, SSI can prolong recovery and delay adjuvant treatment with negative impact on long outcomes [13].

The objective of our study was to differentiate the risk factors for superficial, deep, and organ SSIs after surgeries due to colorectal cancer. Additionally, we put a great emphasis on the method of bowel preparation and its impact on postoperative septic complications. The utilization of MBP and ABX was considered as a likely risk factor of SSIs and other postoperative complications in the early period. We also focused on some groups of risk factors (patient, disease, and treatment specific) and tried to characterize the patterns of SSIs and morbidity with their risk factors for a separate type of SSI.

## Methods

### Patients

From January 2008 to December 2015, all patients who underwent surgery due to colon and rectal cancer were enrolled. We included all surgical interventions (elective and emergency) with resections (i.e., right and left colectomies, resections of the rectum, and abdominal perineal extirpations) and without resections (i.e., explorative laparotomy, bypass, creation of ileostomy or colostomy). The patients were operated on in two colorectal surgical centers. All procedures were performed by staff surgeons among which at least one was an experienced colorectal specialist. We analyzed only open procedures; laparoscopic operations were excluded from the analysis due to their low number in both centers. The study is a retrospective trial, with data collected from records of prospective hospital databases.

### Data collection

The database of patients operated on in the Department of General and Colorectal Surgery Medical University of Lodz (center 1) covered the period between 2008 and 2015, and the database from the Centre for Treatment of Bowel Diseases Hospital in Brzeziny covered the period between 2013 and 2015. The information from these databases was compared according to the outcomes to ensure their consistency.

SSI data were recorded prospectively (according to CDCP) [10] as I—skin superficial, defined as infections of the skin and subcutaneous tissues without involving fascia or muscles; II—deep incisional—infections of fascia and/or muscles in the area of the incision but without any penetration into abdominal cavity; III—organ-space infection—in patients with intraabdominal septic complications (IASC), when any signs of inflammation in peritoneal cavity or pelvic space occurred, including intraabdominal abscesses and anastomotic leaks (ALs). This infection concerned spaces and organs that had to be moved and/or manipulated during the first operation but not the incisional area.

In the postoperative period, a wound surveillance was carried out by four (two in each center) infection-dedicated nurses. After discharge from the hospital, patients were followed up in outpatient clinics by a staff surgeon; any new clinical signs of SSIs were noted and recorded in the database during a 30-day postoperative period. The 30-day follow-up was completed by telephone interview or, if necessary, outpatient visit.

Our study was conducted according to the revised version of the Declaration of Helsinki (October 2008, Seoul). The Local Bioethical Committee gave the consent to carry out the retrospective protocol of the study with the use of prospective records from the hospital databases.

From the hospitals’ databases, successive independent variables were specified: age, gender, obesity (body mass index (BMI)), some biochemical variables, such as level of hemoglobin or albumin concentration, mode of operation (emergency vs. elective), type of surgery (palliative vs. radical), type of resection (with anastomosis vs. without anastomosis), protection of anastomosis in rectal cancer (protective stoma vs. no protection), tumor location (colon vs. rectum, right vs. left colon, upper vs. lower rectum), American Society of Anesthesiologists (ASA) score at admission to the hospital and existing comorbidities, Charlson Comorbidity Index (CCI) [14], preoperative treatment in rectal cancer (radiotherapy and radiochemotherapy), and postoperative staging (based on pathologic report).

Dependent variables: SSIs were divided into three types defined by CDCP [10].

The day before elective surgery, patients (in both centers) underwent MBP (bowel washout with the use of oral macrogol), starting at approximately 2 p.m. together with ABX (p.o. erythromycin 500 mg plus neomycin 500 mg every 4 h, three times: at 1 p.m., 3 p.m., and 8 p.m.), and intravenous antibiotic prophylaxis (metronidazole 500 mg plus cefazolin 1.0 g) was administered directly before incision, irrespective of tumor location (colon or rectal tumor). The intravenous antibiotic prophylaxis was broadened to three doses, if surgery lasted longer than 3 h or in cases of unexpected intraoperative bacterial contamination. Before urgent operations, patients received only intravenous antibiotic prophylaxis, directly before surgery, without MBP and ABX. In the majority of urgent operations, intravenous antibiotic was changed to another one and administered 7 days in the postoperative period.

According to previously published papers in the period from January 2010 to May 2011, the MBP with ABX was abolished. This fact allowed us to build the group of consecutive elective patients with MBP−/ABX−. In our opinion, the early results were not satisfactory; therefore, we have retraced our strategy of MBP+/ABX+ in the next consecutive elective patients since June 2011 until nowadays. In the present study, the effect of MBP−/ABX− on early results was completed with the utilization of the group of patients with the strategy of MBP+/ABX+ (as a comparator), operated on between July 2011 and November 2012.

### Statistical analysis

In the statistical analysis, continuous data were shown as median (range) and differences between compared groups were calculated with the use of *t* tests. Categorical variables were analyzed with the use of a *χ* ^2^ test. We applied a logistic regression model to identify factors associated with the risk of SSIs. Multivariable analysis was presented as the value of adjusted odds ratios (ORs) and corresponding 95 % confidence interval (95 % CI). The differences were considered significant for the level of *p* less than 0.05.

All analyzed clinical factors that likely influenced the occurrence of SSIs were additionally divided into three groups: patient-dependent (age, gender, ASA score, BMI, CCI), diseases-dependent (tumor location, mode of presentation, pathology), and treatment-dependent (type of a surgical procedure, total operation time, the strategy of MBP and ABX, intention of operation (radical, palliative), neoadjuvant therapy, and creation of protective stoma in patients with rectal cancer). In each of these groups, we calculated adjusted OR to assess the risk of occurrence of the particular type of SSI. For statistical analysis, the Statistica Software Version 12.5 (StatSoft, Inc., USA) was used.

## Results

During the study period, a group of 2240 patients (1002, 44.7 % of women; mean age of 67.7) was operated on due to colorectal cancer. Symptoms of SSIs were noted in 364 patients (16.3 %). The incidence of SSIs was similar in women and men (15.3 vs. 17.0 %; *p* = 0.257). In obese patients (BMI >30 pts.), the rate of SSIs was the highest and SSIs occurred in 92 patients (20.1 %), *p* = 0.007. Similarly, within the group of patients with a very poor general condition before operation (IV grade in ASA score), the incidence of SSIs was the highest: 25.2 %, compared with other grades, especially with grade I: 5.8 %, *p* = 0.000.

Taking into account the type of resection, we revealed that abdominal perineal resection was associated with the highest incidence of SSI compared with all other resections. The difference was the largest when compared with right colectomy: 26.4 vs. 13.8 %.

In rectal cancer, the neoadjuvant therapy (radiotherapy and radiochemotherapy) increased the incidence of SSIs 22 and 23.9 vs. 11.9 %; *p* = 0.000, respectively. Protective stoma allowed to obtain significantly lower incidence of SSIs 12.3 vs. 19.1 %; *p* = 0.008. Other details are presented in Table 1.

**Table 1:** Characteristics of patients included in the study: comparison of selected clinical features with the incidence of surgical site infections

	No of patients *n* = 2240 (100.0)	All types of SSIs *n* = 364 (16.3)	*p* value^b^
Gender			
Females	1002 (44.7)	153 (15.3)	0.257
Males	1238 (55.3)	211 (17.0)	
Age			
<65	974 (43.5)	131 (13.4)	0.001
>65	1266 (56.5)	233 (18.4)	
BMI			
<20.0	126 (5.6)	28 (22.2)	
20.01–25.0	772 (34.5)	107 (13.9)	0.007
25.01–30.0	885 (39.5)	137 (15.5)	
>30.01	457 (20.4)	92 (20.1)	
Hgb			
≥12 g/dl	1292 (57.7)	205 (15.9)	0.566
<12 g/dl	948 42.3)	159 (16.8)	
Albumin			
≥35 mg/ml	1478 (66.0)	203 (13.7)	0.000
<35 mg/ml	782 (34.0)	161 (20.6)	
Charlson Comorbidity Index (mean ± SD)	4.5 (±1.1)	7.1 (±1.3)	0.031
Respiratory disease			
No	1595 (71.2)	237 (14.9)	0.005
Yes	645 (28.8)	127 (19.7)	
Cardiovascular disease			
No	1312 (58.6)	198 (15.1)	0.077
Yes	928 (41.4)	166 (17.9)	
ASA Score			
I	103 (4.6)	6 (5.8)	
II	1303 (58.2)	161 (12.4)	0.000
III	679 (30.3)	158 (23.3)	
IV	155 (6.9)	39 (25.2)	
Location of the tumor			
Right colon	498 (22.2)	68 (13.7)	
Left colon	647 (28.9)	107 (16.6)	
Upper rectum	389 (17.4)	61 (15.7)	0.021
Middle rectum	364 (16.3)	53 (14.6)	
Lower rectum	342 (15.2)	75 (21.9)	
Resection procedures			
Right colectomy	413 (21.9)	57 (13.8)	
Left colectomy	171 (9.0)	29 (17.0)	
Sigmoidectomy	282 (14.9)	46 (16.3)	0.014
Hartmann’s	171 (9.0)	23 (13.5)	
AR	309 (16.3)	49 (15.9)	
LAR	381 (20.3)	71 (18.6)	
APR	163 (8.6)	43 (26.4)	
Mode of operation			
Elective	1929 (86.1)	298 (15.4)	0.010
Emergency	311 (13.9)	66 (21.2)	
Anastomosis	1608 (71.8)	287 (17.8)	0.001
Yes	632 (28.2)	77 (12.2)	
No			
Type of operation			
Resection	1890 (84.3)	318 (16.8)	0.086
Palliative only	350 (15.7)	46 (13.1)	
Neoadjuvant therapy^a^			
None	572 (52.2)	68 (11.9)	0.000
Radiotherapy	218 (19.9)	48 (22.0)	
Radiochemotherapy	305 (27.9)	73 (23.9)	
Protective stoma^a^			
Yes	293 (26.8)	36 (12.3)	0.008
No	802 (73.2)	153 (19.1)	
Pathology (AJCC)			
I	417 (18.6)	66 (15.8)	
II	748 (33.4)	119 (15.9)	0.864
III	797 (35.6)	129 (16.2)	
IV	278 (12.4)	50 (18.0)	

Numbers in parentheses are percentages

*SSIs* surgical site infections, *BMI* body mass index, *ASA* American Society of Anesthesiologist, *AJCC* American Joint Committee for Cancer, *AR* anterior resection, *LAR* low anterior resection with the level of anastomosis <6 cm), *APR* abdominal perineal resection

^a^Data refers only to rectal cancer

^b^ *χ* ^2^ test

Septic complications, AL, and 30-day mortality were compared in both centers, and no differences were found. All particulars are listed in Table 2.

**Table 2:** Septic complications and mortality according to analyzed hospitals (centers)

Complication	Centre 1 *n* = 1628 *n* (%)	Centre 2 *n* = 612 *n* (%)	*p* value^a^
All types of SSIs	269 (16.5)	95 (15.5)	0.567
Skin superficial SSIs	173 (10.6)	56 (9.2)	0.304
Deep incisional SSIs	36 (2.2)	15 (2.5)	0.735
Organ space SSIs	60 (3.7)	24 (3.9)	0.793
Anastomotic leak (total)	47 (2.9)	19 (3.1)	
Colon	25 (2.2)	10 (2.5)	0.789
Rectum	22 (3.4)	9 (3.2)	
30-day mortality	20 (1.2)	8 (1.3)	0.882

*Center 1* Department of General and Colorectal Surgery, *center 2* Center for Treatment of Bowel Diseases, *SSIs* surgical site infections

^a^ *χ* ^2^ test

In the group of patients MBP+/ABX+, the incidence of AL was significantly lower than in the group MBP−/ABX−: 2.4 vs. 6.3 %; *p* = 0.008. Organ-space SSIs occurred less frequently in the MBP+/ABX+ group than in MBP−/ABX−: 3.6 vs. 7.2 %; *p* = 0.017. The MBP+/ABX+ did not refer to a lower incidence of all types of SSIs and skin superficial SSIs (Table 3.)

**Table 3:** Type of bowel preparation and the incidence of selected postoperative complications

	MBP+/ABX+ *n* = 291 *n* (%)	MBP−/ABX– *n* = 301 *n* (%)	*p* value^a^
All types of SSIs	46 (15.8)	52 (17.2)	0.631
Skin superficial SSIs	27 (9.3)	18 (6.0)	0.130
Deep incisional SSIs	9 (3.0)	10 (3.6)	0.874
Organ space SSIs	10 (3.6)	24 (7.2)	0.017
Anastomotic leak	7 (2.4)	21 (6.3)	0.008
Obstruction	24 (8.2)	29 (9.8)	0.554
30-day mortality	4 (1.3)	6 (1.9)	0.559

*MBP* mechanical bowel preparation, *ABX* oral antibiotic prophylaxis, *SSIs* surgical site infections

^a^ *χ* ^2^ test

When analyzing selected factors connected strictly with the patient (patient-dependent factors), we noted that age >65 could be a protection for skin superficial and deep incisional SSIs in our group, adjusted OR 0.68 (0.62–0.74 95 % CI) and 0.84 (0.80–0.90 95 % CI), respectively. Obesity was connected with a significantly higher risk of all types of SSIs, adjusted OR 1.53 (1.47–1.59 95 % CI) for skin superficial SSIs and 1.42 (1.39–1.45 95 % CI) for deep incisional SSIs (Table 4.)

**Table 4:** Patient-dependent, disease-dependent, and treatment-dependent factors associated with significantly lower/higher risk of surgical site infections (SSIs) for particular types of SSIs

Factors	All types of SSIs Ad. OR (95 % CI)	Skin superficial SSIs Ad. OR (95 % CI)	Deep incisional SSI Ad. OR (95 % CI)	Organ-space SSIs Ad. OR (95 % CI)
Hospital				
Center 1				
Center 2				
Patient-dependent				
Age				
<65		ref.	ref.	
>65		0.68 (0.62–0.74)	0.84 (0.80–0.90)	
BMI				
<20.0				1.46 (1.38–1.49)
20.01–25.0	ref.	ref.	ref.	ref.
25.01–30.0				
>30.01	1.58 (1.49–1.64)	1.53 (1.47–1.59)	1.42 (1.39–1.45)	1.68 (1.64–1.73)
Albumin				
≥35 mg/ml		ref.	ref.	
<35 mg/ml		1.61 (1.58–1.64)	1.12 (1.10–1.15)	
Respiratory disease				
No		ref.		
Yes		1.29 (1.24–1.33)		
ASA Score				
I	ref.	ref.	ref.	ref.
II				
III		1.12 (1.10–1.14)		
IV	1.82 (1.78–1.83)	1.98 (1.93–2.01)	1.79 (1.75–1.83)	1.80 (1.78–1.83)
Disease-dependent				
Location of the tumor				
Colon		ref.	ref.	
Rectum		1.28 (1.22–1.34)	1.13 (1.09–1.15)	
Location of the tumor				
Right colon	ref.	ref.	ref.	ref.
Left colon				
Upper rectum		0.91 (0.88–0.93)	0.93 (0.90–0.98)	
Middle rectum				
Lower rectum	1.26 (1.22–1.30)	1.34 (1.31–1.36)	1.18 (1.13–1.24)	1.21 (1.18–1.23)
Mode of operation				
Elective	ref.	ref.	ref.	ref.
Emergency	1.34 (1.30–1.39)	1.46 (1.43–1.50)	1.29 (1.23–1.28)	1.32 (1.28–1.34)
Pathology (AJCC)				
I	ref.	ref.	ref.	
II				
III				
IV	1.33 (1.31–1.35)	1.36 (1.33–1.39)	1.29 (1.27–1.32)	
Treatment-dependent				
Type of bowel preparation				
MBP−/ABX−	ref.	ref.	ref.	ref.
MBP+/ABX+				0.53 (0.44–0.59)
Resection procedure				
Right colectomy		0.68 (0.65–0.69)	0.79 (0.77–0.81)	
Left colectomy				
Sigmoidectomy				
Hartmann’s	ref.	ref.	ref.	ref.
AR				1.12 (1.09–1.14)
LAR		1.51 (1.47–1.54)		1.62 (1.60–1.64)
APR	1.69 (1.63–1.75)	1.75 (1.70–1.79)	1.49 (1.43–1.52)	
Anastomosis				
No	ref.		ref.	ref.
Yes	1.77 (1.73–1.82)		1.73 (1.71–1.75)	1.87 (1.83–1.90)
Type of operation				
Palliative		ref.		ref.
Radical		1.39 (1.33–1.43)		1.42 (1.40–1.45)
Operation time				
<180 min	ref.		ref.	ref.
≥180 min	1.64 (1.60–1.71)		1.68 (1.61–1.77)	1.56 (1.48–1.61)
Neoadjuvant therapy^a^				
None	ref.	ref.	ref.	ref.
Radiotherapy	1.72 (1.68–1.77)	1.39 (1.33–1.45)	1.67 (1.65–1.70)	1.78 (1.75–1.80)
Radiochemotherapy	1.64 (1.60–1.69)	1.71 (1.69–1.72)		1.62 (1.60–1.61)
Protective stoma^a^				
Yes			ref.	ref.
No			1.16 (1.13–1.20)	1.13 (1.11–1.16)

Only significant values of adjusted odds ratio (Ad. OR) were presented (*p* < 0.05)

*Center 1* Department of General and Colorectal Surgery, *center 2* Center for Treatment of Bowel Diseases, *MBP* mechanical bowel preparation, *ABX* oral antibiotic prophylaxis, *SSIs* surgical site infections, *BMI* body mass index, *ASA* American Society of Anesthesiologist, *AJCC* American Joint Committee for Cancer, *AR* anterior resection, *LAR* low anterior resection with the level of anastomosis <6 cm), *APR* abdominal perineal resection, *Ad. OR* adjusted odds ratio, *ref*. reference

^a^Data refers only to rectal cancer

Patients with rectal cancer were at a significantly higher risk of skin superficial and deep incisional SSIs than patients with colon cancer, adjusted OR 1.28 (1.22–1.34 95 % CI) and 1.13 (1.09–1.15 95 % CI), respectively. We found that the location of the tumor in the lower rectum was associated with a high risk of all types of SSIs, adjusted OR 1.26 (1.22–1.30 95 % CI), and the highest for skin superficial SSIs, adjusted OR 1.34 (1.31–1.36 95 % CI). When the tumor was located in the upper rectum, the risk of skin superficial and deep incisional SSIs was the lowest, adjusted OR 0.91 (0.88–0.93 95 % CI) and 0.93 (0.90–0.98 95 % CI), respectively (Table 4.)

The group of patients who received MBP+/ABX+ was at a significantly lower risk of organ-space SSIs, adjusted OR 0.53 (0.44–0.59 95 % CI). MBP+/ABX+ had no effect on deep incisional and skin superficial SSIs (Table 4.)

Neoadjuvant therapy (both radiotherapy and radiochemotherapy) increased the risk of all types of SSIs in patients with rectal cancer. The highest risk occurred for organ-space SSIs after radiotherapy, adjusted OR 1.78 (1.75–1.80 95 % CI), and for skin superficial SSIs after radiochemotherapy, adjusted OR 1.71 (1.69–1.72 95 % CI). When patients with rectal cancer underwent abdominal perineal resection (APR), the risk of skin superficial SSIs was the highest when compared with other surgeries, adjusted OR 1.75 (1.70–1.79 95 % CI). The risk of organ-space SSIs was the highest when patients with rectal cancer underwent resection procedure with anastomosis (anterior resection (AR) and low anterior resection (LAR)), adjusted OR 1.12 (1.09–1.14 95 % CI), after AR and even higher after LAR, adjusted OR 1.62 (1.60–1.64 95 % CI). The total operation time longer than 180 min increased the risk of deep incisional and organ-space SSIs (Table 4.)

## Discussion

The colorectal surgery is associated with a very high risk of SSIs as a result of large bacterial load of colon and rectum. SSIs contribute to postoperative morbidity, longer hospital stay [15], and increased hospital costs [16].

Superficial SSIs and organ-space SSI are different postoperative complications with diverse risk factor profiles; therefore, they need to be considered independently. This confirms that we found different risk factor profiles for each type of SSIs.

The large part of our study was devoted to MBP and oral ABX and their impact on the incidence and the risk of SSIs. The primary reason for bowel cleansing is to reduce fecal bulk in order to increase effectiveness of oral antibiotics in the lumen of the colon. Shogan et al. revealed that a standard intravenous antibiotic prophylaxis does not eliminate *Enterococcus faecalis* in the anastomotic tissues and does not prevent AL in animal model [17]; this fact might underline the role of ABX in colorectal surgery. According to the available knowledge, no prospective trials, where patients after colectomy were randomized to receive MBP+/ABX+ or no bowel preparation, have yet been performed. Cannon et al. found that patients who received MBP+/ABX+ before operation revealed a significantly lower incidence of SSIs compared to patients with no bowel preparation [18]. We also hypothesized that patients who receive MBP+ with ABX+ would demonstrate lower rates of SSIs. In recently published studies, authors showed that MBP+ together with ABX+ allowed to reduce the risk of all types of SSIs as well as each type of SSIs separately [12]. In our study, we proved that this strategy was associated with the decreased risk of organ-space SSIs but without the impact on the risk of other types of SSIs. The huge majority of our patients were prepared to surgery with the use of MBP+/ABX+, but the incidence of all types of SSIs was higher than in available literature. We demonstrated lower incidence of AL in patients who received MBP+/ABX+, and this fact stays in accordance with the recent reports [7, 8]. The higher incidence of organ-space SSIs directly resulted from higher incidence of AL. MBP+/ABX+ did not influence the rate of 30-day mortality, which was reported by others [7].

Many recent studies and quality control programs have also focused on assessing the occurrence of SSIs and predicting the risk of SSIs. The incidence of SSIs is approximately 20 %, but the rate was somewhat lower in our study. Nevertheless, SSI develops after large bowel resection and depends on factors associated with the patient, the disease, and the treatment [19–21]. Although many risk factors of SSI were confirmed in several trials, still, there is no consensus on all possible risk factors contributing to SSIs in colorectal cancer surgery [21, 22]. In the present study, we tried to divide all analyzed significant risk factors into risk factors associated with the patient, the disease, and the treatment and connect them with the particular groups of SSI.

Obesity, pulmonary disease, low serum albumin level, and classes III and IV in ASA score were the main patient-dependent factors contributing to skin superficial and deep incisional SSIs. It could be a result of changes in vascularization or poor perfusion of the skin and subcutaneous tissues in these groups of patients. It stays in consistency with other authors [19, 23, 24]. The risk of organ-space SSI was higher in ASA IV and BMI <20 and >30. The awareness implies special surveillance either before or after surgery to avoid the most severe type of SSI. Older age of patients was associated with significantly lower risk of skin superficial and deep incisional SSIs [11]. This set of our results seems to be difficult to explain. Age alone cannot predict early postoperative morbidity, and multiple factors should be evaluated including patients’ functional status and all-known comorbidities. Charlson Comorbidity Index (CCI) is probably the most commonly used index in surgical settings [25]. The CCI gathers all significant patient-dependent factors. We proved that the utilization of CCI might be useful particularly when it amounts to approximately 7.0. We omitted an analysis of predictive values of CCI.

We are not able to change the disease-dependent attributes, but the preoperative recognition of all these factors is crucial. Konishi et al. found patients with rectal cancer as of higher risk of all types of SSI compared with patients with colon cancer [22]. Additionally, in our study, these differences were the most significant in the low rectum, where a considerably large number of patients are needed to be preoperatively irradiated; furthermore, the risk of AL was the highest in the case of these patients [12]. TNM stages III and IV were identified as risk factors of SSI by multivariate analysis [15], but others did not find the stage as a risk factor of incisional SSI [26]. In our study, the incidence of all types of SSIs actually did not differ according to the AJCC stage, but we noted stage IV as liable for a higher risk of skin superficial and deep incisional SSIs. This correlation might result from the extended positivity of lymph nodes and generalized immunological imbalance.

The mode of disease presentation compels the surgeon to perform surgery in urgent course. The operation on patients with peritonitis is associated with a higher risk of wound contamination, predominantly because of the heavy bacterial load of the colon and rectum. Additionally, the preparation to the urgent operation does not allow us to use all components of preoperative antibacterial prophylaxis. In our study, these patients did not receive MBP with ABX, which could bring higher incidence of SSIs.

Preoperative strategies (focused on medical assessment and selection of patients into subgroups with low and high risks of SSIs) would potentially decrease postoperative infection complications. Some authors report disease-related factors as those increasing the risk of organ-space SSI [12, 15, 19]. We did find such associations only for urgent operations and in patients with low rectal tumors.

We noted a higher risk of organ-space SSIs for all assessed treatment-dependent factors. The total operation time, longer than 180 min, independently increased the risk of deep incisional and organ-space SSI, and this finding is in accordance with the latest reports [7, 8]. APR was associated with a higher risk of skin superficial and deep incisional SSIs that could be combined with division of the whole pelvic space and additional perineal wound. It is likely that radiotherapy influenced infections of perineal wounds. Furthermore, the surgery with anastomosis in rectal cancer significantly increased the risk of SSIs, probably because of performing anastomosis in the small pelvis and earlier neoadjuvant therapy.

For the quality improvement programs and initiatives, the knowledge of particular factors that increase the risk of serious (organ-space) SSIs permits the implementation of all available activities to reduce this risk. Some national projects focused on and conducted a series of evidence-based processes of care such as preoperative antibiotic prophylaxis, hair clipping, and intraoperative normothermia [27, 28]. Some organ-space SSIs could conduce to relaparotomy and to all associated implications; in such cases, the process of treatment is usually long and costly.

Recently, two other quality improvement initiatives have been described. Authors used multidisciplinary teams to identify issues in medical care and aimed to reduce the occurrence of SSIs after colorectal surgery. Wick et al. compared rates of SSIs before and after the implementation of a surgery-based comprehensive unit-based safety program (CUSP). In the 12-month period after the implementation of CUSP, they reported a 33 % decrease of the rate of SSIs. CUSP included standardization of skin preparation, antibiotic prophylaxis and bowel preparation, anesthesia, and technique of skin and fascia closure. Cima et al. reported that 1 year after implementation of the National Surgical Quality Improvement Program, the rate of overall and skin superficial SSI was reduced significantly, but it did not influence the rate of organ-space SSIs [29, 30].

We found that different clinical factors contribute to a particular type of SSIs. Our results additionally revealed that in our cohort, the main risk factors of organ-space SSI were strictly associated with the treatment. Considering this fact, the efforts should be directed to each part of the administered therapy and all elements included. Patients of a higher risk should be under special and elaborated surveillance.

We realize some limitations of our study. Firstly, the study is retrospective. The incidence of some skin superficial SSI could be underestimated because patients had to choose another outpatient clinic. The strong side of the study is the large sample size and quite clear homogeneity of the collected data. All analyses were based on two databases conducted in parallel in two specialized colorectal centers. Protocols of the databases were the same, so we could treat the information as quite homogeneous.

## Conclusions

Colorectal resection due to cancer is associated with a high risk of SSIs. If possible, a MBP together with ABX should always be administered to decrease the risk of AL and organ-space SSIs. Clinical factors strictly related to the treatment mostly increased the risk of organ-space SSI in our cohort. All efforts should be undertaken to reduce the risk of all SSIs, however, always taking into consideration each type of SSI separately.
